# 3-Deoxyglucosone: A Potential Glycating Agent Accountable for Structural Alteration in H3 Histone Protein through Generation of Different AGEs

**DOI:** 10.1371/journal.pone.0116804

**Published:** 2015-02-17

**Authors:** Jalaluddin M. Ashraf, Saheem Ahmad, Gulam Rabbani, Qambar Hasan, Arif Tasleem Jan, Eun Ju Lee, Rizwan Hasan Khan, Khursheed Alam, Inho Choi

**Affiliations:** 1 School of Biotechnology, Yeungnam University, Gyeongsan, Republic of Korea; 2 Department of Biotechnology, Integral University, Lucknow, India; 3 Interdisciplinary Biotechnology Unit, Aligarh Muslim University, Aligarh, India; 4 Department of Biochemistry, Jawaharlal Nehru Medical College, Aligarh Muslim University, Aligarh, India; Queen's University Belfast, UNITED KINGDOM

## Abstract

Advanced glycation end-products (AGEs) are heterogeneous group of compounds, known to be implicated in diabetic complications. One of the consequences of the Maillard reaction is attributed to the production of reactive intermediate products such as α-oxoaldehydes. 3-deoxyglucosone (3-DG), an α-oxoaldehyde has been found to be involved in accelerating vascular damage during diabetes. In the present study, calf thymus histone H3 was treated with 3-deoxyglucosone to investigate the generation of AGEs (N^ε^-carboxymethyllysine, pentosidine), by examining the degree of side chain modifications and formation of different intermediates and employing various physicochemical techniques. The results clearly indicate the formation of AGEs and structural changes upon glycation of H3 by 3-deoxyglucosone, which may hamper the normal functioning of H3 histone, that may compromise the veracity of chromatin structures and function in secondary complications of diabetes.

## Introduction

Advanced glycation end products (AGEs) results in the non-enzymatic modifications of biological macromolecules by reducing sugars [1]. The AGE formation process (Maillard reaction) starts with the formation of Schiff bases and the amadori products that are generated in the reaction of reducing sugars with proteins, lipids, and DNA [2–4]. Accumulation of AGEs under *in vivo* conditions has been implicated in secondary complications of diabetes in hyperglycemic environments [5].

Recent studies have suggested that AGEs can also arise from dicarbonyl compounds that are generated from the autoxidation of sugars and other metabolic pathways. It was recently proposed that dicarbonyl compounds could be formed during the glycation process [6]. The accumulation of reactive dicarbonyl compounds termed as carbonyl stress [7], are found elevated (5–6 times) during diabetic complications. Dicarbonyl compounds play an important role in the pathogenesis of diabetic complications since they are highly reactive in modulating the structure and function of intracellular proteins [8]. Reactive dicarbonyl intermediates such as 3-deoxyglucosone (3-DG) and methylglyoxal (MG) are potent precursors of AGEs that contribute significantly to AGEs-formation because they are far more reactive than reducing sugars [9].

Histones are essential components of the eukaryotic chromatin structure. The fundamental structural unit of chromatin, the nucleosome, is formed by wrapping of DNA around histone octamer proteins (H2A, H2B, H3, and H4). The function of histones has been well documented in a wide range of molecular processes; including replication, gene expression, and DNA repair [10]. Histones carry a positive net charge due to the great abundance of arginine and lysine residues in these proteins. N-terminal tails of histones are exposed at the nucleosomal surface, where they become targets for posttranslational modifications [11]. It is well known that histones protect DNA from external damage such as by oxidation. Accordingly, histones may signify the first target for glycation and consequent glycoxidation events in the nuclei to protect the chemical integrity of DNA. However, non-enzymatic glycation damage to nuclear proteins has the potential and severe consequences on maintenance of genomic integrity [12].

To date, there have been no exhaustive or detailed investigations of the glycation of H3 histone protein by 3-deoxyglucosone. Thus, we investigated the glycating potency of 3-DG with H3 histone protein. Physicochemical techniques were employed to characterize the extent of modification of the H3 histone by 3-DG. In addition, formation of various AGEs (Carboxymethyl lysine: CML, pentosidine) and secondary structural changes in H3 histone caused by 3-DG were also investigated.

## Materials and Methods

### Chemicals

Histone protein H3, 3-deoxyglucosone (3-DG), arginine, lysine, sodium azide, 2,4-dinitrophenyl hydrazine (DNPH), 9,10-phenanthrenequinone, dialysis tubing and other reagents/chemicals were acquired from Sigma Chemical Company, USA. NBT was purchased from Sisco Research Laboratories, India.

### Glycation

Calf thymus H3 histone (1 mg/ml) was incubated with 10 μM 3-DG dissolved in PBS (pH 7.4) or in PBS without 3-DG as a control under sterile conditions at 37°C for 14 days. Following incubation, the solutions were extensively dialyzed against PBS to remove excess 3-DG, after which they were stored at -20°C for later use.

### Determination of free amino groups by fluorescamine

The amino groups of native H3 and glycated-H3 samples were estimated by fluorescamine assay as described earlier [13]. Fluorescence of the sample was measured at excitation/emission wavelengths of 390/490 nm using a FLUORO-STAR plate reader (BMG, Germany).

### Determination of free arginine side chains by 9,10-phenanthrenequinone

Free arginine was assayed as previously described [14], after which the fluorescence of the reaction product was measured using a TECAN Saphire spectrophotometer (USA) at excitation/emission wavelengths of 312/395 nm.

### NBT reduction assay

Amadori product (fructosamine) in glycated-H3 was probed by nitroblue tetrazolium (NBT) as previously described [15], after which the absorbance was read at 525 nm and the content of Amadori product (nM/ml) was determined using an extinction coefficient of 12,640 M^-1^ cm^-1^.

### Estimation of protein-bound carbonyl contents

Carbonyl contents of native and glycated-H3 samples were determined using 2,4-dinitrophenylhydrazine [16]. The absorbance was read at 360 nm and the carbonyl content was determined using an extinction coefficient of 22,000 M^-1^ cm^-1^. Protein carbonyl content was expressed as nmol/mg of protein.

### CML content estimation by ELISA

Carboxymethyl lysine (CML) content was measured by 96-well ELISA as previously described [17]. Product formation was measured using a 405 nm filter in an ELX800 multi-well plate reader (BioTek Instruments, USA).

### Chromatographic assay of AGE-CML and pentosidine

CML and pentosidine in the glycated-H3 were estimated by HPLC (High-performance liquid chromatography) as previously described [18]. All enzymatic hydrolysis procedures were performed under nitrogen.

### HPLC

HPLC analysis was conducted to detect the CML and pentosidine in native H3 and 3-DG-glycated-H3 samples after acid hydrolysis as previously described [19].

### *2*.*10*. Spectroscopic analysis

The ultraviolet absorption profiles of native and 3-DG-glycated H3 histone were recorded in the wavelength range of 200–400 nm at 280 nm using a Perkin Elmer Lambda 25 spectrophotometer (Perkin Elmer, Inc., USA) [20].

### Fluorescence analysis

Fluorescence emission profiles of native and 3-DG-glycated H3 histone were acquired using a Hitachi Spectrofluorometer (F-4500; Perkin Elmer, Inc., USA). Samples were excited at 335 and 365 nm and emission profiles were recorded [21]. Increases in the fluorescence intensity (FI) were calculated using the following equation:

$$\%increaseinFI=\left[\left(FI_{glycatedH3}–FI_{nativeH3}\right)/FI_{glycatedH3}\right]\times 100$$

### Circular dichroism (CD) measurements

Far-UV CD profiles of samples were recorded on a Jasco spectropolarimeter (J-815, Japan) attached to a Jasco Peltier-type temperature controller (PTC-424S/15) at wavelengths of 200–250 nm. Control and glycated histones solutions (20 μM) were recorded in 1 mm pathlength cells. Furthermore, the thermal unfolding of native and glycated-histones was conducted as previously described [21].

### FT-IR spectroscopy

Changes in glycated-H3 structure were analyzed by FTIR spectroscopy. Transmission profiles were recorded at 600–4000 cm^-1^ on a Spectrum 100 FTIR spectrometer (Perkin Elmer, Inc., USA).

## Results and Discussion

The glycation processes that lead to AGEs generation have been categorized as biological incidents in the pathogenesis of diabetes complications [22]. Several α-dicarbonyl compounds are involved in glycation reactions and have been linked with hyperglycemia induced damage in diabetes [23]. 3-DG itself causes certain biological activities, including the induction of apoptosis and suppression of cell proliferation [24]. Upon examination of the efficacy of α-oxoaldehydes reactions with proteins, we attempted to discern the 3-DG glycating capability with nuclear protein histone H3.

**Fig 1:**
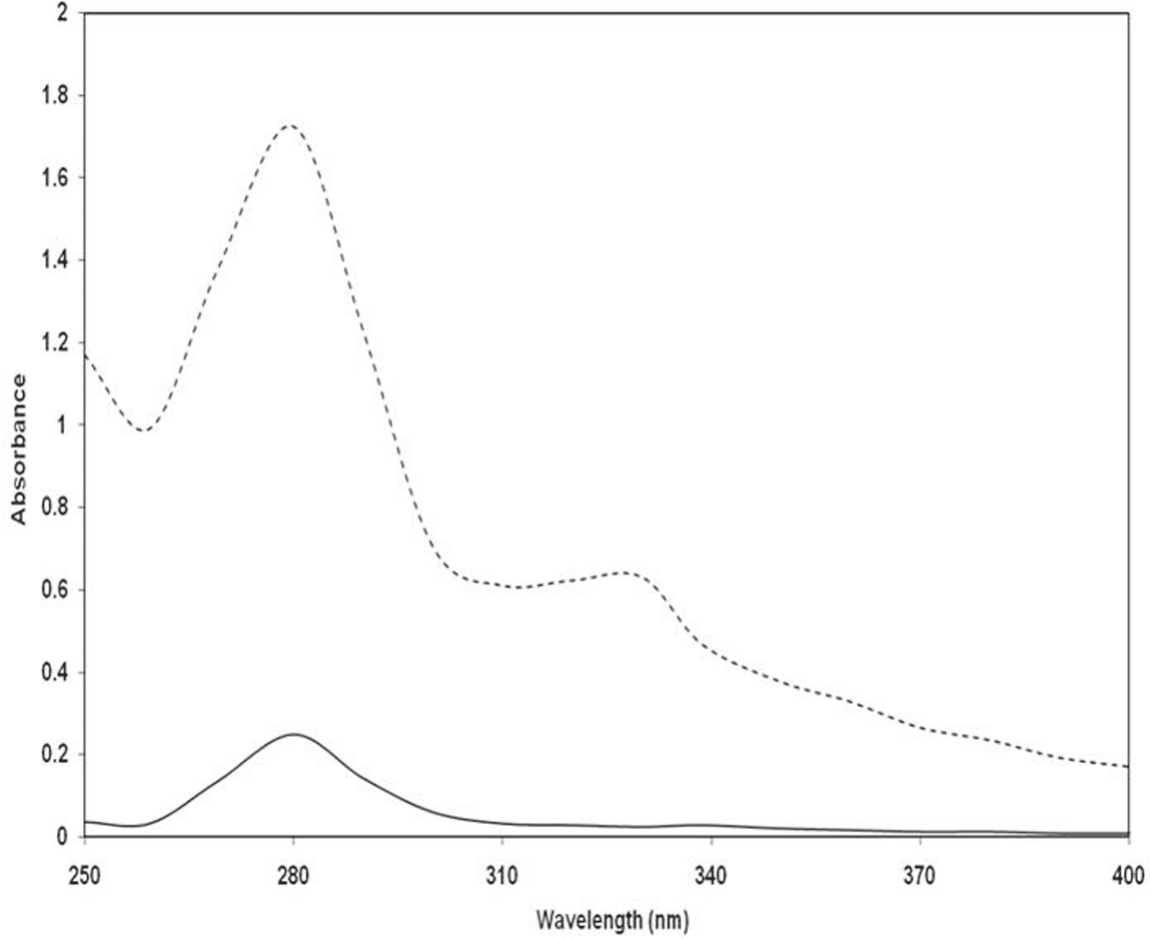
UV-Vis spectral analysis of native (---) and 3-DG-glycated (- - - -) H3 histone.

The percentage of reacted lysine residues in glycated-H3 was 90.75% relative to native H3 (control). Similarly, 83.80% of arginine residues reacted in glycated-H3 when compared to the control (Table 1). The reacted amino acids may predict the extent of glycation and formation of AGEs. A detailed study of 3-DG modified H3 was conducted to detect specific products such as Amadori products and the carbonyl content. Elevated levels of Amadori product (17.03±1.12 nM/ml) were recorded in 3 day glycated-H3 during the early stage of the reaction (Table 1). Earlier studies have revealed that reduction of NBT was due to degradation intermediates of Amadori products [25]. The NBT reduction assay is used solely for determination of Amadori products, and not for AGEs [15]; as Amadori products are markers for the early stage of the glycation reaction [18]. In this study, a considerable amount of carbonyl (26.43±1.21 nM/mg of H3), a known biomarker of oxidative stress, was detected in glycated-H3, suggesting that AGEs formation had occurred (Table 1). Assessment of carbonyl content is useful for measurement of the irreversible oxidative modifications in proteins that occur during glycation [26]. Thus, the production of carbonyl content and Amadori products, as well as side chain modifications of H3 in this system show the glycation reaction between H3 and 3-DG. It has been revealed that 3-DG induces AGEs formation in macromolecules, and investigation of glycated-H3 based on the UV absorption spectra revealed remarkable augmentation of the absorbance (hyperchromicity) at 280 nm [27]. The glycated-H3 showed 85.50% hyperchromicity relative to the native H3. In addition, glycated-H3 showed augmented absorbance in the range of 300–400 nm (Fig. 1). The hyperchromicity recorded at 280 nm may be ascribed to the formation of new chromophoric groups and/or exposure of aromatic amino acids resulting from unfolding of the protein helix upon glycation. Increased absorbance in the range of 300 to 400 nm indicated the formation of AGEs. Several studies have ascribed the hyperchromicity of protein to glycation [28]. In addition, numerous studies have explained the usefulness of increased absorbance between 300 and 400 nm for determining the formation of protein-AGEs [29,30]

**Table 1:** Side chain modifications, formation of various intermediates and AGEs.

Sample	Lysine reacted (%)	Arginine reacted (%)	Carbonyl nmol/gm of H3	CML nmol/mol of H3 (ELISA)	CML nmol/mol of H3 (HPLC)	Pentosidine nmol/mol of H3	Amadori product nM/ml
Native H3	-	-	4.58±0.43	0	0	0	0
3-DG-glycated H3	90.75	83.80	26.43±1.21	2.67±0.16	2.86±0.09	2.53±0.07	17.03±1.12

**Fig 2:**
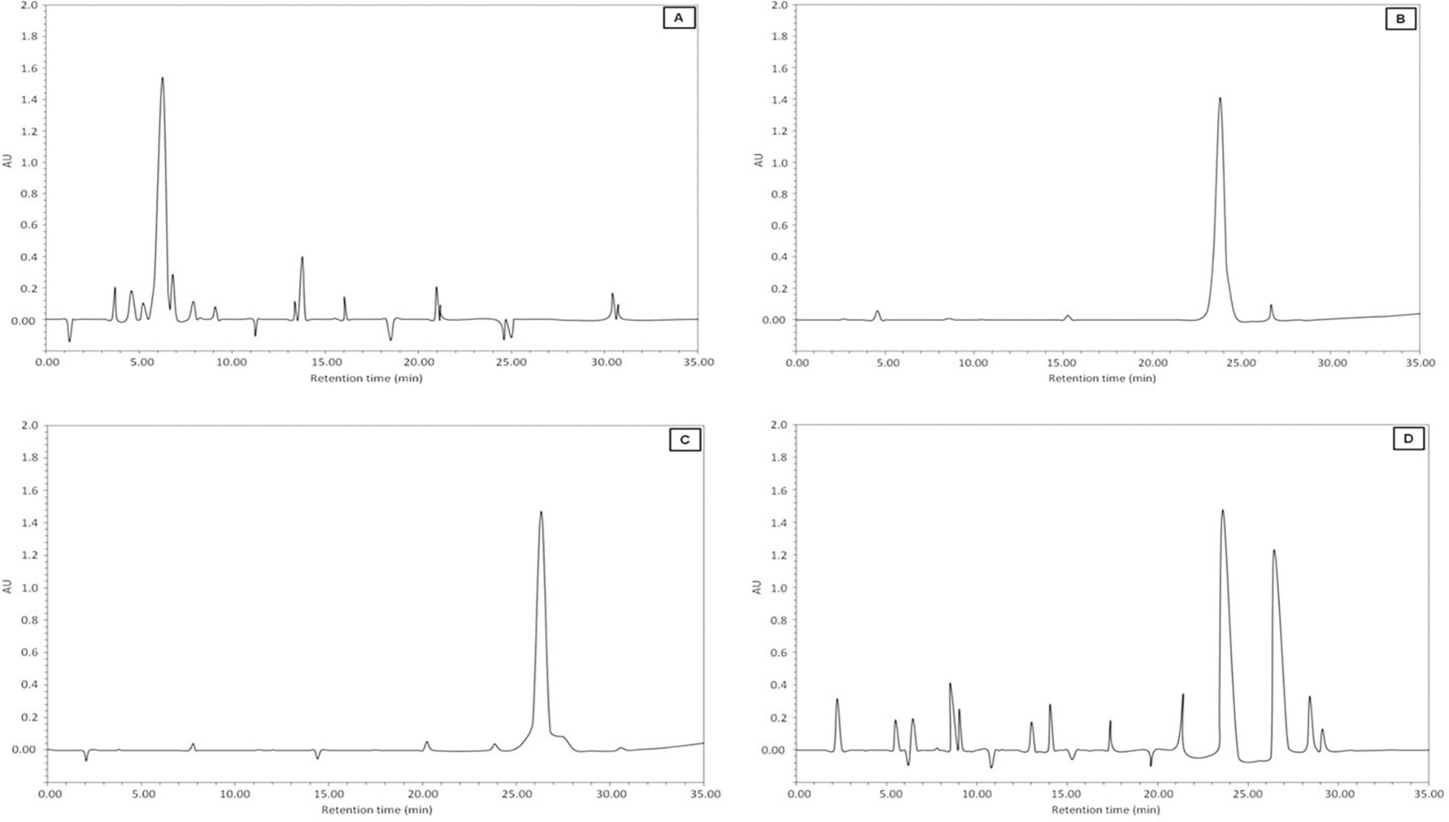
HPLC analysis of native and 3-DG-glycated H3 histone; (A) Chromatogram of native H3, (B) & (C) standard CML and pentosidine, and (D) chromatogram of glycated-H3, respectively.

CML and pentosidine content in glycated-H3 were determined by HPLC assay. Enzymatically hydrolyzed samples demonstrated significant amounts of CML and pentosidine in glycated-H3. The contents of CML and pentosidine in glycated-H3 were 2.86±0.09 and 2.53±0.07 nmol/mol-H3, respectively. Additionally, the CML content determined by CML ELISA confirmed the formation of CML (2.67±0.16 nmol/mol of H3) in glycated-H3 (Table 1). To double check the formation of CML and pentosidine, acid hydrolyzed samples of native and 3-DG-glycated H3 were analyzed by HPLC. The HPLC profiles of standard CML and pentosidine were used as references to confirm the formation of CML and pentosidine in native and 3-DG-glycated H3 samples. The chromatogram of native H3 histone demonstrated no peaks corresponding to CML or pentosidine (Fig. 2A). The standard CML and pentosidine showed distinct peaks with a retention time of approximately 24.20 min and 26.10 min, respectively (Fig. 2B&C), whereas the HPLC chromatogram of the 3-DG-glycated-H3 sample showed two prominent peaks with retention times of 23.30 min and 26.50 min (Fig. 2D), corresponding to standard CML and pentosidine, respectively. HPLC analysis clearly demonstrated the formation of CML and pentosidine in 3DG-glycated H3 histone.

**Fig 3:**
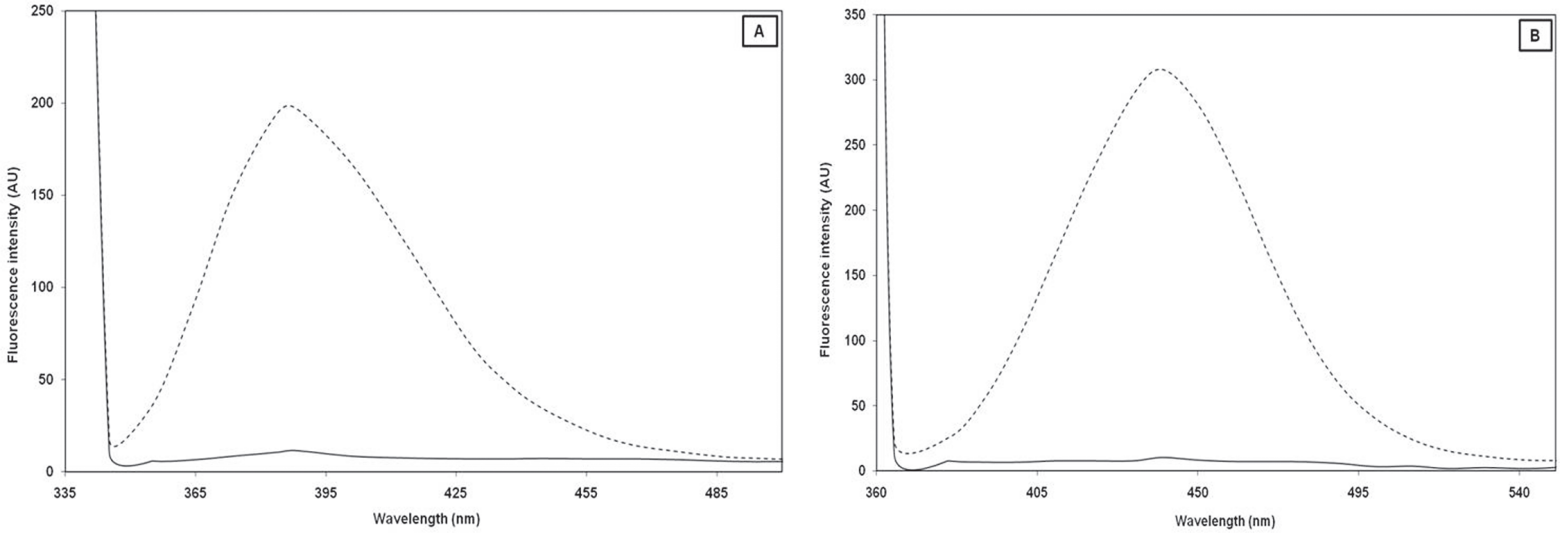
Fluorescence spectral analysis of native (---) and 3-DG-glycated (- - -) H3 histone excited at, (A) 335 nm, and (B) excited at 365 nm respectively.

A fluorescence study was conducted to confirm the generation of fluorogenic AGEs. Previous studies have revealed that a number of fluorogenic AGEs, including pentosidine, are formed during the glycation process [31]. When native and glycated-H3 samples were excited at 335 nm, a 94.17% increase in emission intensity at λ_max_ 388 nm was observed relative to the native H3 (Fig. 3A). Increases in the fluorescence intensity of glycated entities around this excitation/emission wavelength indicate the formation of pentosidine [32]. The high content of lysine and arginine (11–14%) in H3 favors the formation of pentosidine; as crosslinking between arginine and lysine residues is essential for the formation of pentosidine during the glycation reaction [33,34]. Similarly, when the same sample was excited at 365 nm, an approximately 96.55% increase in the fluorescence intensity of glycated-H3 was observed at λ_max_ 440 nm relative to native H3 (Fig. 3B). Since not all AGEs are fluorogenic in nature, excitation at a particular wavelength is indicative of excitement of a particular group or mixture of compounds.

The fluorescence study revealed that pentosidine was formed as one of the AGEs in the glycation reaction between 3-DG and H3 histone. The high fluorescence intensity of glycated-H3 at an emission wavelength of 440 nm indicates that other AGEs were generated when excited at 365 nm. The change in fluorescence intensity upon changing excitation wavelengths suggests the presence of multiple AGEs in the glycated-H3 sample [35]. CML is considered as a marker of protein glycation end products [25], and the results of this study suggest that CML and pentosidine are essential markers for the presence of 3-DG-glycated proteins.

**Fig 4:**
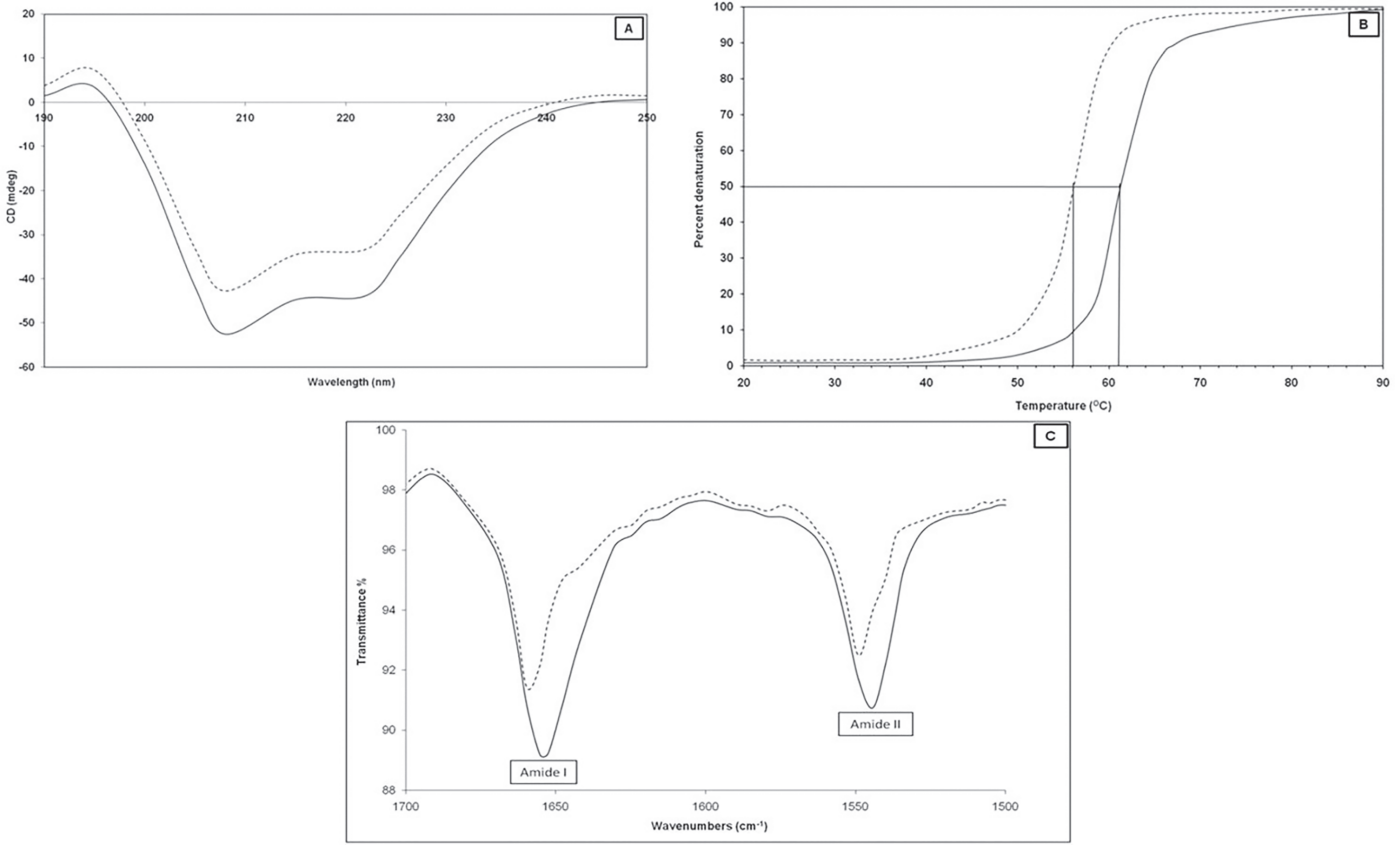
CD spectral, thermal denaturation and FTIR profile of native (---) and 3-DG-glycated (- - - -) H3 histone; (A) Far-UV CD profiles, (B) thermal denaturation showing changes in ellipticity at 222 nm, and (C) FTIR profiles of Amide I and Amide II bands corresponding to native H3 histones and 3-DG-glycated H3 histone.

Keeping in view, the reactivity of 3-DG with H3, the characteristic far-UV CD properties of protein were utilized to assess the secondary structure of H3 proteins and subsequent changes after their modification with 3-DG. Proteins rich in α-helix generate negative bands at 208 and 222 nm. The glycated-H3 histone showed changes in ellipticity at 208 and 222 nm relative to their non-glycated counterparts. 3-DG-glycated H3 demonstrated considerable increases in ellipticity or diminishing CD signal at 208 and 222 nm (Fig. 4A). These events suggest a decrease in the α-helical content, thereby indicates that conformational/secondary structural changes were caused by the interaction of 3-DG with H3 [36,21]. However, the shape of peaks remained almost the same in the presence of 3-DG glycated H3, indicating that glycated H3 has a predominantly α-helix form in nature, even after binding to 3-DG. Decreases in ellipticity indicate that H3 lost α-helix structures during glycation owing to the formation of AGEs, and that consequent conformational changes primarily impair protein functionality by changing protein structure and stability.

The effects of glycation on the thermal stability of native and glycated-H3 were also investigated by carrying out thermal unfolding, which follows the changes in ellipticity at 222 nm. The secondary structure of H3 remained intact at temperatures up to 40°C, while further increases in temperature caused a gradual decrease in CD at 222 nm, showing distortion of the secondary structure. This evidence supports the existence of a rigid and compact structure up to 40°C with strong intra-molecular interactions between the side chains of constituent amino acids [21]. The mid-point temperature (T_m_) of native H3 was 61.25°C, while glycated-H3 showed a substantial decrease in T_m_ to 56.14°C (Fig. 4B). The decrease in the mid-point temperature (T_m_) of glycated-H3 suggests a decrease in thermostability, which is indicative of a decrease in the stability/or loss of secondary protein structure caused by the interaction of 3-DG with H3 protein. These findings demonstrate that some of the interactions stabilizing the secondary structure are disturbed in glycated protein [37].

It is well known that glycation reactions and the resultant AGEs can permanently alter protein structure and function [38]. Most proteins adopt a compact native structure that is energetically stabilized by the presence of several types of atomic interactions within the protein core. Therefore, any physical or chemical phenomena that can disrupt these forces will trigger protein structural changes. The thermal denaturation susceptibility of protein is one of the most fundamental biophysical properties that become altered in proteins [39]. It is important to note that the thermostabilizing effects could be caused by a decrease in the protein's isoelectric point (pI) due to alteration of the surface positive charges, leading to decreased protein stability [40]. In short, lysine and arginine are the most susceptible residues for glycation, forming intra-and intermolecular cross-links between lysine and arginine residues and impairing protein stability. CML and pentosidine formed on side chains of lysine and arginine residues eliminate the positive charge of lysine and arginine residues, affecting thermostability and leading to possible conformational/structural changes and consequently the function of H2A upon glycation.

FT-IR spectra between the 1500–1700 cm^-1^ region reveal two transmittance bands, amide I (C = O bond) and amide II (N-H bond coupled with N-H bending mode). The position of the amide I (1600–1700 cm^-1^) and II (1500–1600 cm^-1^) bands in the FT-IR spectra of proteins are sensitive indicators of conformational changes in the protein secondary structure [41]. Native H3 showed two transmittance bands, amide I at 1654 cm^-1^ and amide II at 1543 cm^-1^ (Fig. 4C). Upon glycation with 3-DG, the positions of the bands and an increase in transmittance were recorded. When compared to the 1654 cm^-1^ (amide I) and 1543 cm^-1^ (amide II) band positions of native H3, the glycated-H3 showed bands at 1660 cm^-1^ (amide I) and 1549 cm^-1^ (amide II). These shifts in band positions and increases in transmittance intensities confirm the perturbation of the secondary structure of H3 histone upon modification by 3-DG [42–44].

Overall, the results of the present study indicate that 3-DG is a reactive compound that generates AGEs and alters the secondary structure of H3. Since H3 is a core histone protein in the nucleosome structure, glycation of H3 may compromise expression of the gene, which could in turn worsen diabetic complications. Thus, 3-DG can be deleterious to biological macromolecules (DNA, proteins, and lipids), especially in disease conditions.
